# Bioactive Components of *Lycium barbarum* and Deep-Processing Fermentation Products

**DOI:** 10.3390/molecules28248044

**Published:** 2023-12-12

**Authors:** Xiao Qiang, Ting Xia, Beibei Geng, Man Zhao, Xuan Li, Yu Zheng, Min Wang

**Affiliations:** State Key Laboratory of Food Nutrition and Safety, Tianjin University of Science and Technology, Tianjin 300457, China; qiangxiao0907@163.com (X.Q.); 15835681506@163.com (B.G.); zhaoman1017@163.com (M.Z.); lixuanlxxx@163.com (X.L.); yuzheng@tust.edu.cn (Y.Z.)

**Keywords:** *Lycium barbarum*, bioactive components, health benefits, fermentation technology, deep-processing products

## Abstract

*Lycium barbarum*, a homology of medicine and food, contains many active ingredients including polysaccharides, polyphenol, betaine, and carotenoids, which has health benefits and economic value. The bioactive components in *Lycium barbarum* exhibit the effects of antioxidation, immune regulation, hypoglycemic effects, and vision improvement. Recently, the development of nutrition and health products of *Lycium barbarum* has been paid more and more attention with the increase in health awareness. A variety of nutrients and bioactive components in wolfberry can be retained or increased using modern fermentation technology. Through fermentation, the products have better flavor and health function, which better meet the needs of market diversification. The main products related to wolfberry fermentation include wolfberry fruit wine, wolfberry fruit vinegar, and lactic acid fermented beverage. In this review, the mainly bioactive components of *Lycium barbarum* and its deep-processing products of fermentation were summarized and compared. It will provide reference for the research and development of fermented and healthy products of *Lycium barbarum*.

## 1. Introduction

*Lycium barbarum*, also known as wolfberry, has both medicinal and edible effects due to its high medical and health care value. Wolfberries are planted in the arid to semiarid environments of Eurasia, Africa, and North and South America [1]. The clean tillage and intercropping with Gramineae are used to cultivate wolfberry, which can increase productivity and sustainability in modern agriculture [2,3]. A total of 31 out of 97 wolfberry species have been recorded to be used as both food and medicine all over the world [4]. Different natural environments, such as the difference in diurnal temperature, rainfall amount, soil thinness, and salinity, lead to different bioactive ingredients [5]. *Lycium barbarum* is placed in the Chinese Pharmacopoeia and Organic Product Certification Catalogue, containing a variety of bioactive components such as polysaccharides, flavonoids, phenolic acids, betaine, and carotenoids [6,7]. Wolfberry recorded in Chinese medicine has the effect of protecting the liver and brightening the eyes, nourishing the kidney and generating essence, dispelling diseases and longevity [8]. It has been confirmed in modern medicine that the overall potency of *Lycium barbarum* includes anti-tumor, anti-inflammatory, and promoting gut health in cell or animal levels [9,10,11].

In recent years, the demand of health products was raised with people’s health awareness increasing. The health value of *Lycium barbarum* was paid great attention, which drove the development of the processing industry. Nowadays, the processing amount of *Lycium barbarum* accounts for one-eighth of its total output. Fresh wolfberry fruits soften rapidly in the ripening period, and then they are stored by drying. And fresh fruits are pressed to retain the concentrated juice for use in the production of various drinks [1,3]. Along with the popularity of wolfberry fruits, they have been transformed into globally traded commodities. In China, wolfberry products are sold that must comply with the Local Food Safety Standard—Goji Berry and the General Hygienic Specification for Food Production [12,13]. In the United States, wolfberries are sold as dietary supplements. They are also widely sold in the EU market as food ingredients. Modified atmosphere packaging and the plant growth regulator such as methyl jasmonate are used to package wolfberry, which minimize the loss of quality and nutritional value in the storage period [14]. The development of deep-processing-related products of wolfberry is still not enough. Therefore, it is urgent to improve the development of the wolfberry industry using modern science and technology.

Modern fermentation technology can not only prolong food storage time, but improve the quality and health value of wolfberry products, which can better meet the needs of modern markets. In this review, the active ingredients of wolfberry and their functions were overviewed, and the main wolfberry fermentation of deep-processing products was further summarized to provide an important reference for the development of the wolfberry health industry.

## 2. Active Ingredients

### 2.1. Lycium barbarum Polysaccharide

*Lycium barbarum* polysaccharide (LBP) is the main active ingredient in *Lycium barbarum*. The total sugar content of *Lycium barbarum* accounts for about 40% of its dry weight, and LBP accounts for 5–8% of the total dry weight. The extraction methods mainly include solvent extraction, enzymatic extraction, and ultrasonic extraction [15]. The solvent extraction method is convenient in operation and low in cost. However, it takes a long time and the extraction rate is limited, and using a high temperature for a long time will lead to polysaccharide degradation and reduce the biological activity. In recent years, enzymatic extraction of polysaccharides has been widely used, and commonly used enzymes include amylase and cellulase [16]. This method has the advantages of environmental protection, high efficiency, mild conditions, and low energy consumption. However, the enzymes are easily disturbed by many influencing factors, such as enzyme type, enzymolysis temperature, and enzyme concentration [17]. Microwave-assisted extraction is a kind of physical technology with low cost, short extraction time, and high extraction rate [18]. Ultrasonic-assisted extraction is a rapid extraction process and retains the structure of bioactive substances contained in natural extracts [19]. Therefore, this method has higher efficiency and lower solvent consumption. Zhang et al. determined the content of LBPs using the phenol-sulfuric acid method, and the results showed that the extraction rates of polysaccharide via hot water extraction and microwave–ultrasonic synergistic extraction were 6.71% and 9.62%, respectively [20]. Xia et al. extracted LBPs using the amylase method and determined its content using the phenol-sulfuric acid method. After optimization via the response surface method and orthogonal test, it was found that the optimal conditions were an enzymolysis temperature of 49.56 °C, enzyme concentration of 0.3%, and enzymolysis time of 140 min. The highest polysaccharide extraction rate was 13.25% [21]. Quan et al. used combination methods of ultrasound and microwave to extract LBPs. The results showed that the optimal conditions of LBP extraction using combined methods were 16 min for microwave extraction, 20 min for ultrasound extraction, and liquid–solid ratio of 55:1. Under these optimized conditions, the polysaccharide yield was 1.873% [22].

In recent years, LBPs have been widely studied, and its molecular weight was ranged from 8 to 214 kDa. Many studies reported that different molecular weights of LBPs had different functions including immunomodulation, antioxidant, and hypoglycemic [23,24,25]. Feng et al. researched LBPs using water extraction and alcohol precipitation, and molecular weights were divided into >10 kDa and <10 kDa after ultrafiltration. The results showed that LBP > 10 kDa induced cell polarization and significantly improved the activity of macrophage RAW264.7 cells. In addition, LBPs upregulated nitric oxide, tumor necrosis factor-α, interleukin-6, and ROS in RAW264.7 cells. The results suggested that LBPs had immunomodulatory effects [25]. Another study reported that LBPs were separated by ultrafiltration membrane separation, and five types of LBPs (LBP1, LBP2, LBP3, LBP4, and LBP5) were obtained with different molecular weights (3, 8, 40, 350, and 400 kDa). The results showed that LBPs inhibited the activity and proliferation of hepatoma H22 cells, and LBP3 with medium molecular weights had the strongest effect in inhibiting H22 liver cancer cells [26]. Yang et al. found four main polysaccharide components (LBLP I, LBLP II, LBLP III, and LBLP IV) via the DEAE-Sephadex A-25 column, with molecular weights of 55.2, 94.0, 241.3, and 418.0 kDa, respectively. LBLP IV improved significantly the lipid profile by upregulating ABCA1 and down-regulating the protein levels of SREBF1 in human pancreatic SW1990 cells compared with three other polysaccharide components. LBLP IV reduced the insulin resistance and raised insulin secretion. It also rose the high-density lipoprotein cholesterol and reduced the blood ester and malondialdehyde. LBLP IV had significant anti-diabetic activity on gestational diabetes mellitus [27]. The composition and function of LBPs are shown in Table 1.

### 2.2. Polyphenols

Polyphenols in *Lycium barbarum* can be found in its leaves, fruits, and root bark including flavonoids, phenolic acids, and proanthocyanidins. Islam et al. determined the contents of total phenols and total flavonoids in red and black wolfberries using colorimetry. The average contents of total phenols were 3.16 and 8.235 mg GAE/g, respectively, and the average contents of total flavonoids were 2.83 and 11.07 mg GAE/g, respectively. The results indicated that the contents of total phenols and total flavonoids in black wolfberry were higher than those in red wolfberry [35]. Ebeydulla found that the total phenols and total flavonoids in *Lycium barbarum* after wax removal and hot drying were 73.98 ± 5.10 and 63.10 ± 5.10 mg GAE/100 g, respectively. The total phenols and total flavonoids obtained via natural air drying were 76.95 ± 2.28 and 66.78 ± 8.84 mg GAE/100 g, respectively. The results suggested that the contents of total phenols and total flavonoids in wolfberry were higher after natural air drying than those after hot drying [36]. Ilić et al. determined the contents of total phenols and total flavonoids in red, black, and yellow wolfberry. Total phenol contents were 162.4 ± 11.5, 176.3 ± 13.0, and 295.7 ± 18.8 mg GAE/100 g, and total flavonoid contents were 214.2 ± 28.6, 335.5 ± 27.1, and 27.40 ± 4.76 mg HE/100 g, respectively. The results showed that the content of total phenols was the highest in yellow wolfberry, and the content of total flavonoids was the highest in black wolfberry [37]. Taken together, the content of polyphenols in *Lycium barbarum* is influenced by various factors, including its types and drying methods.

The detection methods of polyphenols in *Lycium barbarum* mainly included high-performance liquid chromatography (HPLC), gas chromatography–mass spectrometry (GC–MS), and ultra-performance liquid chromatography–triple quadrupole mass spectrometry (UPLC–TQ-MS). Flavonoids were identified as rutin, quercetin, kaempferol, myricetin, hesperidin, and naringin. Phenolic acids included chlorogenic acid, p-coumaric acid, ferulic acid, protocatechuic acid, and gallic acid. Liu et al. reported the characterization of the eleven phenolic compounds in *Lycium barbarum* using the liquid chromatography–high-resolution mass spectrometry (LC-HRMS/QTOF) method, including chlorogenic acid, esculetin, caffeic acid, rutin, ellagic acid, p-hydroxycinnamic acid, scopoletin, 5,7-dihydroxy-4-methylcoumarin, morin, quercetin, and curcumin. Phenolic compounds demonstrated not only decreased ROS levels and matrix metalloproteinase expressions, but also strengthened intrinsic antioxidant defense systems including superoxide dismutase, glutathione peroxidase, and catalase in the UV-irradiated mice. Therefore, the phenolic compounds in *Lycium barbarum* resulted in the improvement of skin collagen content and skin barrier dysfunction [38]. A recent study showed that five main phenolics were detected and further identified using HPLC–MS analysis, including rutin, p-coumaric acid, and its hexoside derivatives. These components were verified to have antioxidant activity by employing three in vitro antioxidant assays, including 1,1-diphenyl-2-picrylhydrazyl (DPPH), 2,2′-azinobis (3-ethlybenzothiazoline)-6-sulfonic acid (ABTS), and the ferric ion reducing antioxidant power (FRAP). The correlation between antioxidant capacity and five main phenolics ranging from high to low was ABTS, FRAP, and DPPH [39]. The types, contents, and detection methods of compounds contained in *Lycium barbarum* polyphenols are shown in Table 2.

Proanthocyanidins (PAs) existing in monomer or oligomeric/polymeric form are known as condensed tannins and have antioxidant, anti-inflammatory, and anti-aging effects [48,49]. The content of PAs in wolfberry was different in different varieties, pulps, and treatment methods. Its extraction methods were similar to polysaccharide extraction methods, such as organic solvent extraction, enzyme extraction, and ultrasonic-assisted extraction [50,51,52]. Islam et al. determined the contents of PAs in red and black wolfberry using colorimetry. Black wolfberry had the highest content of condensed tannins, which was 23.51 mg CAE/g, while red wolfberry had the lowest content, which was 0.86 mg CAE/g [35]. Xin et al. determined the content of PAs in the pulp and seeds of four black wolfberries using colorimetry. The average content of PAs in wolfberry pulp was 19.57 mg CAE/g and that in seeds was 2.03 mg CAE/g. The average content of red wolfberry pulp was 18.85 mg CAE/g and that of seeds was 0.35 mg CAE/g. The findings suggest that PA content in black wolfberry was higher than that in red wolfberry, and PA content in pulp was higher than that in seeds [53]. Lu et al. determined the content of PAs in wolfberry using the butanol chlorination method. The content of PAs ranged from 2.98 to 9.15 mg PAE/g dw after sun drying, hot-air drying, and freeze-drying. And the PAs in wolfberry via freeze-drying were the highest among the three drying methods [54]. By comparing the above studies, PAs had the highest content in the black wolfberry and in wolfberry pulp. Additionally, PAs were detected using the freeze-drying method with the highest content, which will provide a theoretical foundation for the subsequent development and research of polyphenols in *Lycium barbarum*.

### 2.3. Betaine

Betaine belonging to alkaloids is measured in *Lycium barbarum* as a quality standard. At present, the detection methods of betaine mainly include HPLC, ion chromatography, ultra-performance convergence chromatography–tandem mass spectrometry (UPC2-MS), thin-layer chromatography (TLC), and colorimetry. TLC and colorimetry are greatly influenced by external factors, which have low sensitivity. Ion chromatography has the advantages of continuity, high efficiency, and sensitivity, but the detection time is too long. HPLC has the characteristics of high efficiency, high speed, high sensitivity, wide application range, and low cost, so HPLC is the most commonly used detection method. Moreover, UPC2-MS is a new technology with high sensitivity and selectivity. Compared with HPLC, it has faster separation speed and better efficiency, but a higher cost.

A recent study showed that the content of betaine was determined using high-performance liquid chromatography–diode array detection (HPLC–DAD), and the result showed that the content of betaine was 1.45–1.65% [55]. Yan et al. demonstrated betaine in wolfberry using the ion chromatographic–pulsed amperometric method (IC–PAD). The results suggested that the betaine content decreased after processing, and the content of betaine in dried wolfberry fruit was 7.49 g/kg and that in wolfberry juice was 1.52 g/kg [56]. In addition, Zhang et al. determined betaine content in *Lycium barbarum* from 11 producing areas using UPC2-MS. The results showed that the contents of *Lycium barbarum* from Zhongning were the highest (1.39 ± 0.08 μg/g). The content of wolfberry Jiuquan had the lowest, which was 1.20 ± 0.21 μg/g [57]. Therefore, different growth conditions of wolfberry in different regions, such as growth time, picking time, temperature, and soil, led to differences in betaine content in wolfberry.

Recent researchers showed that betaine in wolfberry had many important biological characteristics, including antioxidation, anti-inflammatory, and hypoglycemic effects [58,59,60]. Yang et al. found that betaine (400 mg/kg) reduced the expression levels of NF-κB, TNF-α, and IL-1β proteins in the lungs of rats with monocrotaline-induced pulmonary arterial hypertension. The results showed that betaine decreased inflammatory reaction and inhibited vascular remodeling by down-regulating the NF-κB signaling pathway and improving the role of pulmonary hypertension [61]. Additionally, Chien et al. found that betaine in wolfberry had important effects in alleviating dry eye disease. The Schirmer’s test score and tear break-up time were significantly increased, and the severity of the keratoconjunctival staining was decreased significantly by betaine on dry eye disease in rats [62]. Therefore, the betaine, as a functional active ingredient of *Lycium barbarum*, has health benefits and provides the scientific basis for the development of wolfberry functional food.

### 2.4. Carotenoids

Carotenoids exist in the form of free or ester, and the color of *Lycium barbarum* is determined by the content of carotenoids, which can be divided into red wolfberry, black wolfberry, and yellow wolfberry according to different colors [63]. Mi et al. analyzed the carotenoid content in red and yellow wolfberries using colorimetry. The results showed that the content of total carotenoids in red wolfberry was higher than that in yellow wolfberry, which was 39.74–54.76 mg/100 g, and its content was directly proportional to the redness of wolfberry [64]. Islam and others determined the carotenoid content using colorimetry and found that the highest carotenoid content in red wolfberry was 233.08 µg/g. The content of black wolfberry was the lowest, which was 1.51 µg/g. Therefore, the carotenoid content was followed in the order of red wolfberry, yellow wolfberry, and black wolfberry [35].

In recent years, researchers detected the types and contents of carotenoids in wolfberry. Ren et al. detected carotenoids in wolfberry using HPLC combined with one test and multiple evaluations. The results showed that zeaxanthin, β-carotene, and zeaxanthin dipalmitate were obtained. Among them, the content of zeaxanthin dipalmitate was high, ranging from 0.81 mg/g to 4.05 mg/g. Secondly, zeaxanthin was 5.88–28.17 μg/g. The content of β-carotene was close to the limit value [65]. Bai et al. detected the carotenoids of wolfberry from five different habitats using HPLC. The results showed that there were three components, namely zeaxanthin dipalmitate, zeaxanthin, and β-carotene. Zeaxanthin dipalmitate had the highest content, reaching 21.03 mg/mL, followed by zeaxanthin, and the content of β-carotene was the lowest, which was 0.01 mg/mL [66]. Aneta et al. used liquid chromatography–quadrupole-time-of-flight mass spectrometry (LC–QTOF-MS) to detect carotenoids in *Lycium barbarum*. Four components were identified as zeaxanthin, β-carotene, neoxanthin, and cryptoflavin. The content of zeaxanthin was the highest with an average of 845.39 mg/kg. The lowest was neoxanthin with an average content of 160.35 mg/kg [67]. Hsu et al. detected nine components in the carotenoid extract of wolfberry using HPLC, including all-trans zeaxanthin and its three isomers, all-trans β-carotene and its two isomers, neoxanthin, and β-cryptoxanthin. The results showed that the highest content of all-trans zeaxanthin and its three isomers was 1666.3 μg/g and the lowest content of neoxanthin was 4.47 μg/g [68]. The detection methods of carotenoids in *Lycium barbarum* are shown in Table 3.

In addition, the carotenoids extraction significantly inhibited the growth of colon cancer cells by upregulating the levels of p53 and p21 and down-regulating the levels of CDK2, CDK1, cyclin A, and cyclin B in vitro. It was found that *Lycium barbarum* was the main source of zeaxanthin, which was particularly important for preventing and improving vision in early age-related macular degeneration (AMD) [73]. Li et al. randomly selected 114 patients with early AMD and supplemented them with 25 g of wolfberry every day for 3 months. The results showed that the patient’s zeaxanthin level increased and the optical density of macular pigment increased. The findings suggested that zeaxanthin in wolfberry improved the vision of AMD patients [74].

### 2.5. Other Ingredients

In addition to the above active ingredients, *Lycium barbarum* also contains a variety of amino acids, vitamins, trace elements, and other ingredients, which play an important role in maintaining the health of the body. Wang et al. determined the amino acid content in wild wolfberry via amino acid analyzer. A total of 16 kinds of amino acids were detected, and aspartic acid content was the highest (3.00 mg/g) [75]. Zhou et al. determined the amino acid content of red, black, and yellow wolfberries using amino acid analyzer. The results showed that the amino acid content of black wolfberry was higher than the other two types, which reached 9.04 g/100 g. The aspartic acid content was 0.37–1.30 g/100 g, which was the highest among 17 amino acids. The contents of magnesium, calcium, iron, manganese, copper, and zinc in black wolfberry were detected. The iron content was the highest (36.1 mg/100 g), followed by zinc (10.4 mg/100 g) [76]. Justyna et al. determined trace elements in wolfberry using size exclusion chromatography coupled to inductively coupled plasma mass spectrometry (SEC-ICP-MS). These included manganese, iron, copper, zinc, selenium, and molybdenum in wolfberry fruit. Among them, the content of zinc was the highest (10.6 μg/g) The contents of manganese and copper were 9.9 μg/g and 6.1 μg/g, respectively [77].

*Lycium barbarum* is also rich in vitamins, including vitamin A (VA), vitamin B1 (VB1), vitamin B2 (VB2), and vitamin C (VC). Aneta et al. determined the content of VC in 21 kinds of wolfberry using the UPLC–PDA method, and the content was 2.39–6.24 mg/100 g. The g43 had the highest content and g22 had the lowest in 21 kinds of wolfberries [67]. Another study reported VC in two kinds (Lasa and New Big) of *Lycium barbarum* planted in Lithuania via titration, and the highest concentration of vitamin C was found in the New Big (5.8 mg/100 g FW) [78]. Yang determined the content of VB1 in cultivated wolfberry and wild wolfberry using the fluorescence method. The average contents were 7.87 μg/g and 7.10 μg/g, respectively, and the content of cultivated wolfberry was higher than that of wild growth, which may be due to the good growth environment and sufficient water supply [79]. Zhang et al. analyzed the contents of VB1 and VB2 in black wolfberry and red gijo berry using the UPLC method. The contents of VB1 in black and red wolfberries were 21.7 mg/kg and 9.4 mg/kg, respectively. The content of VB2 was 17.6 mg/kg and 4.5 mg/kg, respectively. The content of VB in black wolfberry was higher than that in red wolfberry [80]. The contents of VA and VE were detected in black wolfberry oil using HPLC, and the contents were 0.3 mg/100 g and 46.3 mg/100 g, respectively [81].

## 3. *Lycium barbarum* Deep-Processing Products of Fermentation

**Figure 1:**
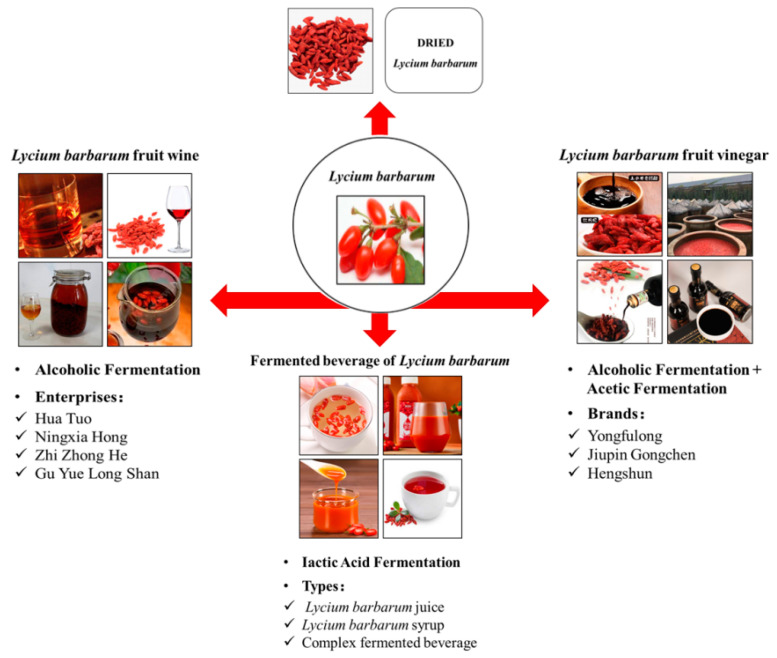
*Lycium barbarum* fermentation deep-processing products.

As the wolfberry contains a variety of active functional ingredients, the medical health care function of wolfberry has been gradually paid attention to in recent years. Recently, with people’s health care awareness increasing, the market demand for wolfberry products needs to be expanded continuously. However, the fresh wolfberry fruit is seasonal, has a short harvesting period, and is easy to decay and deteriorate; therefore, it is difficult to preserve and needs to be processed in time to extend the shelf life. China is the most important place for plant, production, and consumption of wolfberry. At present, 80% of wolfberries are sold as primary processed products of dried fruits [82]. The products are relatively single in the market, and deep-processing products are less. The *Lycium barbarum* fermentation deep-processing product and its characteristics are shown in Table 4. The primary processing products of wolfberry had low technological content products, insufficient market recognition, and backward processing equipment, which restricted the development of the modern industry of wolfberries [83,84]. Therefore, deep-processing products are needed to research and develop. Due to the lower nutrients loss and more active ingredients brought by fermentation, the fermented deep-processed products of wolfberry can be better exploited and promote the technological progress of the wolfberry industry. The wolfberry deep-processing products via fermentation are shown in Figure 1.

### 3.1. Wolfberry Fruit Wine

Wolfberry wine can be divided into fermented type and mixed type according to different production processes. Fermented wolfberry wine is made by adding yeast to raw materials through alcohol fermentation, which is one of the modern wines of health care [85]. At present, wolfberry wine mainly focuses on developing the new type of wolfberry wine. The production technology of wolfberry wine is stable, and there are representative wolfberry wine enterprises such as Hua Tuo and Ningxia Hong.

During the fermentation process, maceration plays an important role in extracting compounds from the fruit skin into the wine, and these compounds after the maceration process can be metabolized, producing better flavor and taste during the fermentation and aging processes. A study reported that wolfberry wine was macerated via short-time maceration (SM, 48 h), middle-time maceration (MM, 72 h), and long-time maceration (LM, 96 h) during alcohol fermentation. The contents of flavor substances in the MM wine were highest. In addition, esters and ketones were higher in the MM wine compared with those in SM and LM, which provide the better flavor and fruity aroma for the wine. The composition of aromatic compounds is mainly determined by the fruit genotype and maceration, and the appropriate maceration time is the key factor to enhance aromatic compounds and the sensory attributes of wine [86]. Another study reported that wolfberry juice at a concentration of 50 g/L was added into amber ale beer to improve the sensory and nutritional values. The fermented beer was characterized by lower turbidity and higher antioxidant ability and bioactive compounds (rutin and 2-O-b-D-glucopyranosyl-L-ascorbic acid) [87]. Ren et al. studied the optimization for the fermented process of wolfberry wine, which used different maturity levels as raw materials (mature berries, mildly over-matured berries, and severely over-matured berries). Wine with mature berries was the best fermentation product, which was based on critical indicators of nutritional and organoleptic quality. The highest contents of polysaccharides (1050 ± 0.13 mg/L) and total flavonoids (1216.0 ± 2.49 mg/L) were both found in wine with mature berries. It indicated that the quality of wolfberry wine was mainly determined by raw materials, which was agreed to be a primary factor in producing high-quality wolfberry wine [88]. A previous study reported that wolfberry fruit wine had antioxidant properties in vitro, which, determined by DPPH and ABTS trialsm were 1.27–1.87 g/L and 1.61–2.13 g/L, respectively. The antioxidant properties of wolfberry fruit wine were much higher than those of Chinese wine [89]. Although wolfberry wine has high nutritional and health value, the development of wolfberry needs to improve through several aspects, such as improving product quality, reducing production costs, and clarifying product functional attributes.

### 3.2. Wolfberry Fruit Vinegar

The wolfberry vinegar is a kind of sour drink with excellent nutritional value and flavor, which is made using modern production technology. Fruit vinegar is the common fermented beverage known as the fourth generation of beverages. Its taste is sweet and sour and also has the healthy effects of both vinegar and fruit. Due to the raw materials and fermentation, the products contain a variety of nutritional and active components, such as organic acids, amino acids, and polyphenols [90,91]. Some studies have reported that fruit vinegar has the effects of antibacterial and antioxidant activities, anti-aging, and anti-fatigue [92,93,94].

Li et al. reported on the production process of composite fruit vinegar using wolfberry and grapes as the main raw materials. The optimal technological conditions for alcohol fermentation were 6% yeast addition, initial pH of 4.0, fermentation temperature of 28 °C, and fermentation time of 6 days. Under these optimized conditions, the alcohol content reached 8.28%vol. The optimal process conditions for acetic acid fermentation were 4% acetic acid bacteria inoculation, fermentation temperature of 32 °C, and fermentation time of 6 days. Under these optimized conditions, the total acid content was 4.27 g/100 mL, which made the fruit vinegar have pure flavor and special fruit aroma [69]. Another study reported that wolfberry wine was inoculated 10% acetic acid bacteria and fermented for 6 days at 30 °C and 180 rpm, and wolfberry vinegar was brewed. The total phenolic and flavonoid contents in wolfberry vinegar were 2.42 mg GAE/mL and 1.67 mg RE/mL, respectively. The contents of betaine and carotenoid were 2.88 ± 0.22 and 0.42 ± 0.02 mg/mL, respectively. In addition, *Lycium barbarum* polysaccharides were the main bioactive components in WFV with a content of 8.94 ± 0.27 mg/mL. Furthermore, there is a protective effect of wolfberry fruit vinegar on the liver. It was found that wolfberry fruit vinegar treatment effectively alleviated liver injury by inhibiting oxidation levels and increasing antioxidant levels in CCl_4_-treated mice [95]. Taken together, the fermentation process of wolfberry fruit vinegar can improve the utilization rate of raw materials, screen excellent strains, and enhance fermentation efficiency, which are the key factors to control cost. At present, there are less kinds of wolfberry fruit vinegars on the market, such as Haitian, Guanli, and Hengshun. Therefore, wolfberry fruit vinegar needs to further increase research and development to improve market competitiveness and market sales.

### 3.3. Fermented Wolfberry Beverage

In recent years, the fermented beverage has gradually concerned itself with beneficial effects on the human body. At present, most of fermented wolfberry beverages are brewed using lactic acid bacteria, yeast, and acetic acid bacteria [3,96]. The products fermented with single or mixed strains of lactic acid bacteria are mainly occupying the market share of beverages. It has been reported that fermentation can sufficiently use the nutrition of wolfberry, which is an important research direction in the field of deep processing wolfberries [97]. Fermentation technology influences the active ingredients of wolfberry, which is an important factor to determine the quality and function of the products [98]. Current research is mainly focused on the optimization of fermentation process and the influence on the active substances in fermented wolfberry beverages.

Wang et al. reported the effects of four different lactic acid bacteria (*L. brevis strain* CICC 6239, *L. plantarum* strain CICC 6240, *L. acidophilus* strain CICC 22150, and *L. plantarum* strain CICC 23138) on the growth rate and antioxidant activity during the fermentation. The results showed that the *L. brevis* CICC 6239 strain had the best growth rate in wolfberry juice. Total polyphenols and antioxidant activity gradually increased during fermentation, and the *L. acidophilus* strain CICC 22150 had the highest content of total polyphenols (4.23 g GAE/L) and antioxidant activity (81%) at the end of fermentation [99]. Another study reported the effects of six different lactic acid bacteria (*Lactobacillus plantarum* 90, *Lactobacillus casei* 37, *Lactobacillus paracasei* 01, *Lactobacillus acidophilus* 85, *Lactobacillus helveticus* 76, and *Bifidobacterium lactis* 80) on bioactive substances and antioxidant activity during wolfberry juice fermentation. Among the six strains, the total phenols and total flavonoids contents were the highest throughout the *Lactobacillus helveticus* 76 fermentation, which significantly rose from 1460 mg GAE/mL to 1800 mg GAE/mL and 412 mg RE/mL to 530 mg RE/mL, respectively. Additionally, the highest content of rutin was during *Lactobacillus casei* 37 fermentation, which was increased from 23.76 to 100.52 mg/L. *Lactobacillus paracasei* 01 had the strongest ability to increase betaine, the content raising from 8237.77 to 24,529.63 mg/L [100]. In addition, another study reported that fermented wolfberry beverage via *Lactobacillus plantarum*, *Lactobacillus reuteri,* and *Streptococcus thermophilus* exhibited better anti-inflammatory effects than unfermented wolfberry beverages. The fermented wolfberry beverage modulated gut microbiota and attenuated dextran sodium sulfate-induced ulcerative colitis in mice [101]. At present, the wolfberry beverages mainly included wolfberry juice, which occupied most markets. However, there are few types of wolfberry-fermented beverages in the market. Wolfberry-fermented beverages have the advantages of soft taste, unique flavor, and various nutrients, which needs to be further explored to broaden the market of wolfberries.

## 4. Conclusions

*Lycium barbarum* has a long history as a medicinal and functional food. It is rich in various active ingredients such as polysaccharides, polyphenols, flavonoids, and betaine, which have the efficacy of antioxidant, immune regulation, hypoglycemia, and vision improvement. The wolfberry has attracted attention from domestic and international markets in recent years due to their high-quality health value and great development potential. At present, the *Lycium barbarum* fermentation deep-processing products were mainly wolfberry fruit wine-, vinegar-, and lactic acid-fermented beverages. However, wolfberry, as a compound ingredient, is often used in meat products, milk products, and bakery and confectionary products via the continuous development of technology [1,3,102]. There are a few fermented products of wolfberry, which need further development. The trend of wolfberry deep-processed products is based on more natural and functional foods to meet people’s demand for health. The development of wolfberries still needs to be improved from the following aspects: (1) In order to protect sensitive compounds and exhibit better therapeutic effects, new types of nanoliposomes and microcapsules of wolfberry should be developed in the future [103,104]. (2) Further research on fermentation technology could also be integrated into artificial intelligence such as machine learning and artificial neural network. Then, based on the detection of substances such as sugars and proteins during the fermentation process, a warning is given to adjust fermentation parameters in a timely manner to ensure the quality and safety of the products [105,106]. (3) Future works could focus on the health benefit of wolfberry deep-processing products in vivo and in vitro, such as anti-aging, eye protection, and neuroprotection [107,108,109]. However, it is urgently necessary to develop the deep-processing products of wolfberry fermentation in the future to meet the diversified needs of the market.

## Figures and Tables

**Table 1:** Composition and function of polysaccharides in *Lycium barbarum.*

*Lycium barbarum* Polysaccharides	Constitutions	Functions	References
LBP	Arabinose, rhamnose, galactose, galacturonic acid, glucose, glucosamine, xylose, mannose, fructose, ribose	Regulates immunity and inflammation	[25]
LBP3	Arabinose, galactose	To treat Alzheimer’s disease	[24]
LBP1B-S-2	Arabinose, rhamnose, galactose, glucuronic acid	Inhibit tumor angiogenesis	[28]
BBP-24-3	Arabinose, glucose, galactoaluronic acid	Lower blood sugar levels after meals	[29]
p-LBP	Arabinose, rhamnose, galactose, glucose, gluconic acid, galacturonic acid, xylose, fructose	Hypoglycemic effect	[30]
LBP	Arabose, rhamnose, galactose, galactosamine, glucose, gluconic acid, xylose, mannose, ribose	Induced immune response	[31]
LBP	Arabinose, galactose, glucose	Anti-inflammatory, antioxidant, modulating immunity	[32]
PLBP	Arabinose, rhamnose, galactose, galacturonic acid, xylose	Improve immunity, antioxidant	[33]
LBP-4a	Arabose, rhamnose, galactose, glucose, xylose, mannose	Renal protection	[34]

**Table 2:** The compound types and contents and detection methods of polyphenols in *Lycium barbarum.*

Polyphenols	Compound Types and Contents	Detection Methods	References
Flavone	Myricetin (57.2 mg/g), mul-berin (12.7 mg/g), rutin (9.1 mg/g)	HPLC	[40]
	Quercetin (65.33 ± 9.5–369.8 ± 44.5 μg/g), myricetin (53.2 ± 8.9–117.3 ± 4.9 μg/g), kaempferol (15.3 ± 2.8–93.6 ± 6.7 μg/g), quercetin-rhamnose-dihexoside (434.7 ± 6.4–1065 ± 65.3 μg/g), rutin (43.2 ± 8.3–76.1 ± 8.3 μg/g), quercetin-3-*O*-rubutin (158.9 ± 4.3–628.9 ± 21.5 μg/g)	HPLC	[41]
	Rutin (1947 μg/g), naringin (2.03 μg/g), aurantiamarin (9.50 μg/g), neohesperidin (8.49 μg/g), naringenin (52.2 μg/g), hesperetin (1.32 μg/g)	UHPLC–MS/MS	[42]
	Rutin (1323.99 ± 21.13–7229.32 ± 0.12 μg/g), kaempferol-3-*O*-rubutin (69.43 ± 0.58–1066.02 ± 0.44 μg/g)	UPLC–TQ-MS	[43]
	Rutin (143.98 ± 62.1 μg/g), myricetin (4.56 ± 0.15 μg/g), quercetin (4.02 ± 0.12 μg/g), kaempferol (0.78 ± 0.05 μg/g), naringenin (0.98 ± 0.02 μg/g)	UPLC–Q-Orbitrap MS	[44]
Phenolic acid	Neochlorogenic acid (3.13 ± 0.02–574.21 ± 0.25 μg/g), protocatechualdehyde (0.87 ± 0.00–9.47 ± 0.06 μg/g), p-hydroxybenzoic acid (6.79 ± 3.51–190.08 ± 0.85 μg/g), p-coumaric acid (1.64 ± 3.77–67.70 ± 0.27 μg/g), chlorogenic acid (77.07 ± 12.33–10,203.92 ± 1.96 μg/g), caffeic acid (0.58 ± 0.05–19.37 ± 2.55 μg/g), ferulic acid (3.10 ± 0.48–19.55 ± 0.32 μg/g), cryptochlorogenic acid (12.33 ± 2.80–961.93 ± 11.23 μg/g)	UPLC–TQ-MS	[43]
	Gallate (13.5 ± 0.17 μg/g), catechin (5.46 ± 0.13 μg/g), chlorogenic acid (162.66 ± 24.34 μg/g), vanillic acid (2.88 ± 0.08 μg/g), caffeic acid (119.7 ± 21.65 μg/g), syringic acid (1.02 ± 0.01 μg/g), p-coumaric acid (554.4 ± 38.7 μg/g), ferulic acid (114.54 ± 15.7 μg/g), salicylic acid (2.41 ± 0.07 μg/g), gallogen (4.5 ± 0.14 μg/g)	UPLC–Q-Orbitrap MS	[44]
	Chlorogenic acid (6.48 ± 0.16 mg/g), caffeic acid (1.41 ± 0.043 mg/g), syringic acid (0.15 ± 0.01 mg/g), p-coumaric acid (0.83 ± 0.03 mg/g), ferulic acid (1.17 ± 0.04 mg/g)	HPLC	[45]
	Salicylic acid (1.8 ± 0.1–2.3 ± 0.4 ng/mg), 4-hydroxybenzoic acid (7.8 ± 0.1–8.1 ± 0.3 ng/mg), syringic acid (0.3 ± 0.1–0.9 ± 0.1 ng/mg), p-coumaric acid (6.8 ± 0.1–178.4 ± 9.3 ng/mg), vanillic acid (1.8 ± 0.1–26.4 ± 0.3 ng/mg), gallate (1.2 ± 0.1–1.9 ± 0.3 ng/mg), caffeic acid (0.7 ± 0.1–2.5 ± 0.3 ng/mg), protocatechuic acid (0.7 ± 0.1–1.0 ± 0.1 ng/mg), ferulic acid (31.3 ± 0.3–33.6 ± 3.6 ng/mg)	GC–MS	[46]
	Protocatechuic acid (91.6 ± 0.4 ng/g), trans caffeic acid (46.4 ± 0.1 ng/g), gentisic acid (18.2 ± 0.0 ng/g), p-coumaric acid (1644.1 ± 3.5 ng/g), ferulic acid (684.2 ± 2.4 ng/g), isoferulic acid (9120.1 ± 3.1 ng/g), salicylic acid (508.4 ± 2.2 ng/g), hydroxybenzoic acid (664.3 ± 3.2 ng/g)	LC–ESI-MS/MS	[47]

**Table 3:** The compound types and contents and detection methods of carotenoids in *Lycium barbarum.*

Compound Types and Contents	Detection Methods	References
Zeaxanthin (28.17 μg/g), β-carotene (5.62–8.04 μg/g), zeaxanthin dipalmitate (0.81–4.05 mg/g)	HPLC	[65]
Zeaxanthin dipalmitate (21.03 mg/mL), zeaxanthin (0.14 mg/mL), β-carotene (0.01 mg/mL)	HPLC	[66]
Zeaxanthin (845.39 mg/kg), β-carotene (193.53 mg/kg), neoxanthin (160.35 mg/kg), cryptoflavin (722.94 mg/kg)	LC–QTOF-MS	[67]
All-trans zeaxanthin and its three isomers (1666.3 μg/g), all-trans β-carotene and its two isomers (20.11 μg/g), neoxanthin (4.47 μg/g), β-cryptoxanthin (51.69 μg/g)	HPLC	[68]
(3R, 3′S)-zeaxanthin (0.522 µg/mL), (3R, 3′R)-zeaxanthin (0.398 µg/mL), (3R, 3′R, 6′R)-lutein (0.582 µg/mL)	HPLC	[69]
Zeaxanthin (0.6 ± 0.2%), β-carotene (0.8 ± 0.2%), zeaxanthin palmitate (3.4 ± 0.2%), β-cryptoxanthin palmitate (5.1 ± 1.1%), antheraxanthin dipalmitate (1.0 ± 0.2%), zeaxanthin myristate palmitate (1.9 ± 0.4%), zeaxanthin dipalmitatec (80.4 ± 0.6%), zeaxanthin palmitate stearate (1.1 ± 0.1%)	HPLC–PDA-MS	[70]
β-carotene (0.02–7.9 μg/g), lutein (0.2–97.5 μg/g), lycopene (0.1–22.0 μg/g), violaxanthin (0.1–47.7 μg/g), zeaxanthin (0.02–14.2 μg/g), zeaxanthin dipalmitate (0.2–94.2 μg/g)	UPLC–MS	[71]
Zeaxanthin dipalmitate (4.5–5.5 mg/g)	HPLC–DAD	[72]
Zeaxanthin dipalmitate (211.4 mg/100 g), zeaxanthin dipalmitate esters (37.5 mg/100 g), β-carotene (1.2 mg/100 g)	HPLC	[73]

**Table 4:** *Lycium barbarum* fermentation deep-processing products.

Deep-Processing Products	Production Process	Characteristics	Existing Problems
*Lycium barbarum* fruit wine	alcoholic fermentation	High nutrition and health care value	Market positioning deviation, higher pricing
*Lycium barbarum* fruit vinegar	alcoholic fermentation and acetic fermentation	Good taste, nutrition, health care, fashion in one	Market category is small, to be further expanded development
Fermented beverage of *Lycium barbarum*	lactic acid fermentation	Soft taste, unique flavor, rich nutrition	The market is small and the sales are low

## Data Availability

Data sharing not applicable.
